# Correction to: Highlights of the 36th EANM Annual Congress 2023, from hometown Vienna, Austria: “A SYMPHONY OF SCIENCE”

**DOI:** 10.1007/s00259-024-06723-9

**Published:** 2024-04-25

**Authors:** David Kersting, Silvia Morbelli, Sophie E. M. Veldhuijzen van Zanten, Hein J. Verberne

**Affiliations:** 1 Department of Nuclear Medicine and German Cancer Consortium (DKTK) Partner Site, West German Cancer Center (WTZ), University Hospital Essen, University of Duisburg-Essen, Essen, Germany; 2 Department of Medical Sciences, University of Turin, Turin, Italy; 3 Nuclear Medicine Unit, Citta’ della Salute e della Scienza di Torino, Turin, Italy; 4 Department of Radiology and Nuclear Medicine, Erasmus MC, Rotterdam, the Netherlands; 5 Department of Radiology and Nuclear Medicine, Amsterdam UMC, Location AMC, University of Amsterdam, Amsterdam, the Netherlands

**Correction to: European Journal of Nuclear Medicine and Molecular Imaging**

10.1007/s00259-024-06692-z

The article Highlights of the 36th EANM Annual Congress 2023, from hometown Vienna, Austria: “A SYMPHONY OF SCIENCE”, written by David Kersting, Silvia Morbelli, Sophie E. M. Veldhuijzen van Zanten, and Hein J. Verberne, was originally published online on the publisher’s internet portal on April 4, 2024 with Open Access under a Creative Commons Attribution (CC BY) license 4.0. With the author’s/authors’ decision to cancel Open Access the copyright of the article changed to © The Author(s), under exclusive licence to Springer-Verlag GmbH Germany, part of Springer Nature 2024 with all rights reserved.

The original article has been corrected.

